# Protein Contact Map Prediction Based on ResNet and DenseNet

**DOI:** 10.1155/2020/7584968

**Published:** 2020-04-06

**Authors:** Zhong Li, Yuele Lin, Arne Elofsson, Yuhua Yao

**Affiliations:** ^1^School of Science, Zhejiang Sci-Tech University, Hangzhou 310018, China; ^2^School of Information Science and Technology, Zhejiang Sci-Tech University, Hangzhou 310018, China; ^3^Department of Biochemistry and Biophysics, Science for Life Laboratory, Stockholm University, Stockholm, Solna 17121, Sweden; ^4^School of Mathematics and Statistics, Hainan Normal University, Haikou 571158, China

## Abstract

Residue-residue contact prediction has become an increasingly important tool for modeling the three-dimensional structure of a protein when no homologous structure is available. Ultradeep residual neural network (ResNet) has become the most popular method for making contact predictions because it captures the contextual information between residues. In this paper, we propose a novel deep neural network framework for contact prediction which combines ResNet and DenseNet. This framework uses 1D ResNet to process sequential features, and besides PSSM, SS3, and solvent accessibility, we have introduced a new feature, position-specific frequency matrix (PSFM), as an input. Using ResNet's residual module and identity mapping, it can effectively process sequential features after which the outer concatenation function is used for sequential and pairwise features. Prediction accuracy is improved following a final processing step using the dense connection of DenseNet. The prediction accuracy of the protein contact map shows that our method is more effective than other popular methods due to the new network architecture and the added feature input.

## 1. Introduction

Proteins perform a wide range of cellular functions and, in most instances, their function is related to their structure. Experimental determination of protein structure is time-consuming and expensive; therefore, accurate protein structure prediction can play a vital role in understanding protein function. If a protein of interest is homologous to one whose structure has already been determined, it is possible to model the structure using the homologous protein's structure as a template. For many proteins, there are no suitable templates available and it is therefore necessary to develop methods that can use only the amino acid sequence to predict protein structure. It has been shown that the best method to obtain a structure is to determine whether a pair of residues in a protein sequence is in contact.

The current prediction methods used to construct protein contact maps are divided into direct coupling analysis (DCA) methods and machine learning methods. DCA methods utilize multiple sequence alignments (MSAs) to determine the correlation between residue pairs. While this method achieves results when the target protein sequence has many homologous sequences in the protein database, the evolutionary coupling information generates “noise signals.” DCA generally uses graphical lasso and pseudo-likelihood maximization methods to solve this problem [1]. Graphical lasso can estimate the graph structure from the covariance matrix using a likelihood estimation of the precision matrix with L1 regularization. Pseudo-likelihood maximization is an approximate method for probabilistic models to estimate the strength of interactions between residues. Popular DCA methods include CCMPred [2], FreeContact [3], GREMLIN [4], and PSICOV [5]. While these methods are useful for constructing protein contact maps when a high number of sequence homologs are available, their accuracy is poor when the number of homologs is low.

Machine learning methods have been widely used to make various protein predictions and can perform better than DCA methods when fewer homologous sequences are available because they can learn sequence feature relationships when given a labelled dataset. The first machine learning methods used support vector machines (SVM) [6] and other related methods such as SVMCon [7] and R2C [8], due to their capacity to construct classification models. With the development of artificial neural networks, deep learning methods (including various forms of recurrent neural networks [9] and deep belief networks [10]) have become mainstream frameworks for biological prediction programs including Betacon [11], CMAPPro [12], DeepConPred [13], NNCon [14], and MetaPSICOV [15]. RaptorX-Contact [16] and DNCON2 [17] are recently released methods and are the approaches that attempt to use the entire protein image as a context prediction. It should be noted that all methods, except for DEEPCOV [18], use one or several DCA methods as their inputs.

RaptorX-Contact [16] is one of the state-of-the-art contact predictors. Its high accuracy, as confirmed by CASP [19, 20], demonstrates the benefit of acquiring the whole protein for use as a context for constructing a contact map. It applies the ResNet structure for the prediction which can solve the problems of gradient disappearance and explosion due to its identity and residual mapping characteristics, but the number of parameters is proportional to its depth. DNCON2 [17] divides its predictor into two parts. The first uses a series of intermediate convolutional neural networks to predict the contact map at five distances (6~10 Å), and the second combines these separate predictions into another convolutional neural networks to provide a final contact map at 8 Å. The PconsC4 method [21] is one of the latest contact map prediction methods which is composed of ResNet [22] and U-net [23] network structures. It can capture 1D and 2D protein features to predict the contact map. However, the feature map size from the U-net network differs between input and output and is therefore inconsistent before and after downsampling. This means that upsampling data cannot be restored entirely before downsampling, which may have a negative impact on prediction accuracy.

In this paper, we present an integrated deep learning network-based approach to predict a protein contact map. We trained the network framework using a protein training dataset with known structures then tested it on public datasets including the CASP [19, 20], CAMEO [24], and membrane protein [25] datasets. In our method, sequential and pairwise features are used as input for the network structure. For the sequential features, besides sequence profile (PSSM), predicted protein secondary structure (SS3) and solvent accessibility (ACC), we introduce a new position-specific frequency matrix (PSFM) feature which we have found complements the PSSM features. The pairwise features include direct coevolution information generated by CCMPred, mutual information from MSA, and pairwise potential [26]. Fusion of these features effectively represents the features of the protein sequence necessary for protein contact prediction. The deep learning framework used in our method is composed of ResNet and DenseNet. This network structure fully integrates the advantages of identical mapping and residual mapping of ResNet with the dense connection of DenseNet, so that the network depth of our method is not too deep, it can effectively reduce gradient disappearance, enhance feature transmission, and to a certain extent, reduce the number of parameters. DenseNet's input and output feature map formats remain the same, which allow for greater feature retention and thus improve the accuracy of predicted protein contact map. Our experimental results show that our method yielded better accuracy than other popular methods.

## 2. Models and Methods

### 2.1. Datasets

The dataset used is a subset of PDB25, extracted from the PDB database (http://www.rcsb. org/pdb/home/home.do), in which the sequence identity of any two proteins is less than 25%. Proteins satisfying any one of the following conditions were excluded (1) sequence length is less than 26 or greater than 700 amino acids, (2) resolution is less than 2.5 Å, and (3) has multiple protein chains. To eliminate redundancy in the dataset, we exclude any proteins which had >25% sequence identity which left us with 6767 proteins. We randomly chose 6000 proteins to train the model and used the remaining proteins to validate it. To evaluate our method, we used the widely used and publically available hard target datasets, CAMEO [24], Mems400 [25], and CASP12 and 13 [19, 20]. In these test sets, the sequence identity between any two protein sequences was less than 25%.

### 2.2. Contact Map Definition

If two residues are in contact in the protein contact map [12] means that the Euclidean distance between the two C*β* atoms of the residues (glycine is a C*α* atom) is less than 8 Å. Contacts are divided into three categories based on the separation between the two residues: (1) long-range contacts, when the separation is greater than 24 residues; (2) medium-range contacts, when the separation is between 12 and 23 residues; and (3) short-range contacts, when the separation is between 6 and 11 residues.

**Figure 1:**
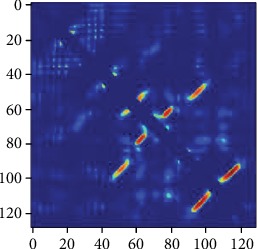
Protein contact map.

A protein contact map example is shown in Figure 1. It illustrates the probability of contact between the two residues. The horizontal and vertical coordinates represent the protein sequence, with colored dots indicating the probability of contact between the two residues (range between 0 and 1, the redder the color, the higher the possibility of contact).

### 2.3. Feature Extraction

In this method, there are two types of protein features, one-dimensional (sequential feature) and two-dimensional (pairwise feature), which are used in the prediction of the protein contact map. The one-dimensional feature includes sequence profile (position-specific scoring matrix, PSSM), 3-state protein secondary structure (SS3), and 3-state solvent accessibility (ACC). The 20-dimensional position specific scoring matrix (PSSM) [27] is obtained by searching the uniprot_sprot database (ftp://ftp.uniprot.org/pub/databases/uniprot/current_ release/knowledgebase/complete) with HHblits [28] to generate sequence profiles with three iterations and *E*-values set to 0.001. The 3-state protein secondary structure is taken from Bi-LSTM [29], and the 3-state solvent accessibility is taken from DSPRED [30]. Two-dimensional features include direct coevolution information generated by CCMPred, mutual information from the multiple sequence alignment (MSA), and pairwise potential [26].

**Figure 2:**
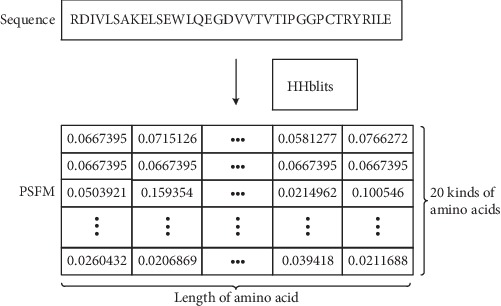
Representation of PSFM feature.

Because the position-specific frequency matrix (PSFM) [31] contains the frequency of amino acids in the protein sequence, we can add it to the input feature to complement the advantages of the position-specific scoring matrix. Here, we ran the HHblits [28] program, with three iterations and *E*-values set to 0.001, to search the uniprot_sprot database to generate MSA, and then calculate PSSM and PSFM based on the HHblits results. With *L* = protein sequence length and *n* = feature dimension, PSSM is represented by a two-dimensional matrix of L × 20, the secondary structure is represented by a two-dimensional matrix of L × 3, the solvent accessibility is denoted by a two-dimensional matrix of L × 3, and the PSFM is represented by a two-dimensional matrix of L × 20 (Figure 2). The one-dimensional feature of our method is then expressed by a two-dimensional matrix of L × 46. The two-dimensional feature is represented by a three-dimensional matrix of L × L × 5. The element in the PSFM matrix is the target frequencies, which represents the occurrence frequency of one amino acid at a specific position in the protein sequence in the evolutionary process. The sum of the frequencies in each line is 1.

### 2.4. Prediction Model

We propose a new integrated deep learning method to map predicted protein contacts composed of residual neural network (ResNet) [22] and densely connected convolutional networks (DenseNet) [32] which form a neural network framework.

#### 2.4.1. Residual Neural Network

**Figure 3:**
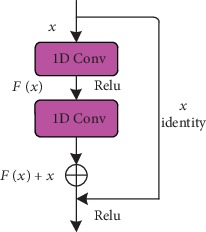
Residual learning: a building block.

The residual neural network (ResNet) consists of a residual learning model (Figure 3) which can be defined as:

(1) $$\begin{array}{c} y=F\left(x,\left\{W_{i}\right\}\right)+x, \end{array}$$

where *x* and *y* are the input and output vectors of the layers to be considered, *W*_*i*_ is the weight in the weight matrix, and *F* represents the residual mapping to be learned. For an example that has two layers (Figure 3), its residual mapping function is as follows:

(2) $$\begin{array}{c} F=W_{2}f\left(W_{1}x+b_{1}\right)+b_{2}, \end{array}$$

(3) $$\begin{array}{c} f\left(x\right)=\max\left(0,x\right), \end{array}$$

where *f* denotes the Rectified Linear Unit activation function, and *W*_1_, *W*_2_, *b*_1_, and *b*_2_ are the weights and biases of the first layer and the second layer, respectively.

#### 2.4.2. Densely Connected Convolutional Networks

**Figure 4:**
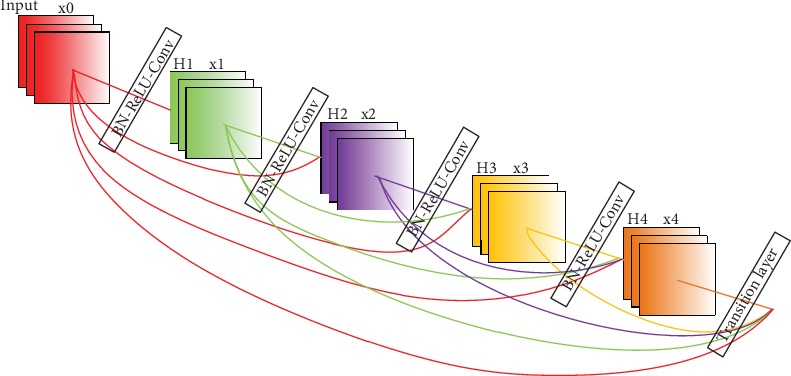
Dense block connections of DenseNet.

Compared with the convolutional neural network and other deep learning methods, the residual neural network can, to a certain extent, solve the problems of gradient descent and disappearance. The number of residual neural network parameters is proportional to its depth and because each layer has independent weights, when the number of layers increases, the number of parameters does too. Fortunately, this problem can be effectively solved by the densely connected convolutional networks (DenseNet). DenseNet consists of a dense block, a transition layer, and a bottleneck layer. The dense block (Figure 4) is composed of an *l*-layer network and a composite function. The composite function consists of a normalization function, linear rectification unit, and convolution function. The *l*-layer of its *l*^th^ layer network has *l* inputs, that is, the *l*th layer receives all the outputs of feature maps from the previous *l* − 1 layer. Its construction formula is as follows:

(4) $$\begin{array}{c} X_{l}=H_{l}\left(\left[X_{0},X_{1},\cdots ,X_{l-1}\right]\right), \end{array}$$

where [*X*_0_, *X*_1_, ⋯, *X*_*l*−1_] means to connect the feature map from layer 0 to layer *l* − 1.

The transition layer is composed of convolution and pool layers. The bottleneck layer consists of a 1 × 1 convolution which is used to reduce the number of feature maps and improve calculation efficiency.

#### 2.4.3. Integrated Model

**Figure 5:**
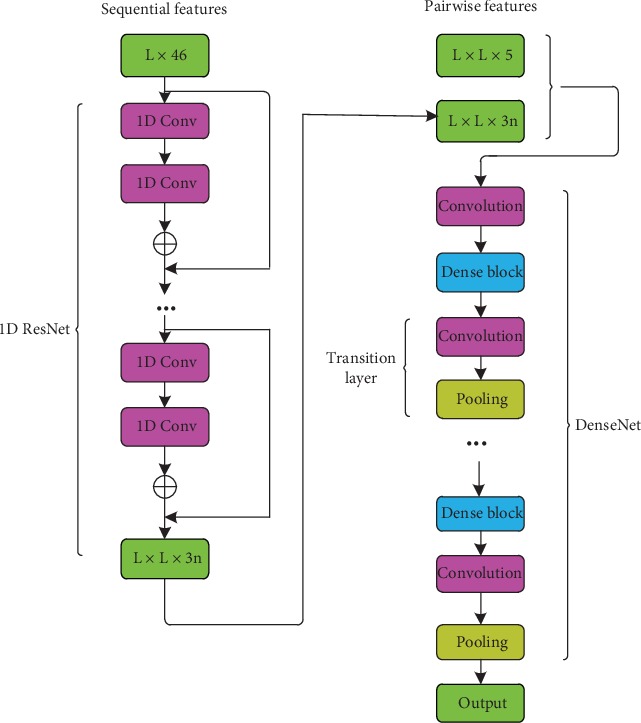
Integrated model framework.

Our framework is based on an integrated deep neural network (Figure 5) and is composed of one-dimensional residual and densely connected convolutional networks. ResNet can, to a certain extent, solve the problems of gradient disappearance and explosion due to its identity and residual mapping characteristics and can train the deep network structure, but the number of ResNet parameters is proportional to its depth. DenseNet can effectively reduce the problem of gradient disappearance due to its dense connection characteristics and it can, to a certain extent, reuse features, thus strengthening feature transfer and reducing parameter numbers. DenseNet retains the input and output feature map formats, so it can maintain features as much as possible.

In summary, we have integrated two kinds of network structures (ResNet (Figure 3) and DenseNet (Figure 4)) and made use each one's advantages to improve the accuracy of the predicted protein contact map. In the data preparation stage of our framework, sequential features (one-dimensional features) are represented by a vector of L × 46, which are sent into the ResNet network. Then, pairwise features (two-dimensional features) are combined with one-dimensional features from one-dimensional residual network, and all of them are sent into the DenseNet network.

In ResNet, there are several residual learning models, each of which is composed of two convolution layers with convolution kernel size of 3. After each convolution layer, there is a Rectified Linear Units activation function [33], and then an outer concatenation function [16] is applied to convert the output results from two-dimensional to three-dimensional. Namely, let *v* = {*v*_1_, *v*_2_, ⋯, *v*_*i*_, ⋯, *v*_*L*_} be the final output of the first module where *L* is the protein sequence length and *v*_*i*_ is a feature vector storing the output information for residue *i*. For a pair of residues, *i* and *j*, we concatenate *v*_*i*_, *v*_(*i* + *j*)/2_, and *v*_*j*_ into a single vector and use it as one input feature of this residue pair. We then combine them with pairwise features to form the input for the second part of the network. To prevent the network from overfitting, we utilize a dropout algorithm with an 80% dropout ratio to randomly discard neurons during training. We used an effective stochastic optimization method using the gradient descent optimization and set the learning rate as 0.01. In our model, the maximum likelihood function is used to train the model parameters, and the loss function is defined as a negative log-likelihood function, namely, the cross-entropy function. The formula is

(5) $$\begin{array}{c} E\left(t,y\right)=-\underset{i}{\sum }t_{i}\log y_{i}, \end{array}$$

where *t*_*i*_ is the label and *y*_*i*_ is the predicted result.

### 2.5. Performance Evaluation

The results can be divided into four categories: true positive (TP), false negative (FN), false positive (FP), and true negative (TN). TP refers to a positive group samples that are correctly predicted, FN refers to a positive group that is incorrectly predicted to be negative, FP refers to a negative group that is incorrectly predicted to be positive, and TN refers to a correctly predicted negative group. Based on these indexes, we use the following evaluation criteria to predict the performance of our method and compare it to other methods.

Precision refers to the proportion of the correct number of positive samples in the total number of samples determined by the classifier to be positive.

(6) $$\begin{array}{c} \text{Precision}=\frac{\text{TP}}{\text{TP}+\text{FP}}. \end{array}$$

Recall refers to the proportion of the correct number of positive samples in the actual number of positive samples.

(7) $$\begin{array}{c} \text{Recall}=\frac{\text{TP}}{\text{TP}+\text{FN}}. \end{array}$$

F1 score is the harmonic mean of the precision and recall.

(8) $$\begin{array}{c} \text{F}1=2∙\frac{\text{Precision}∙\text{Recall}}{\text{Precision}+\text{Recall}}. \end{array}$$

## 3. Experimental Results

In our experiment, we use top *L*/*k* (*k* = 10, 5, 2, and 1) in the long-range contact to evaluate prediction accuracy of the protein contact map. *L* is the length of the sequence, and prediction accuracy rates are given in three kinds of contact. To verify our model's validity, we tested our prediction accuracy on the PDB25, CAMEO, and Mems400 datasets (http://raptorx.uchicago.edu/contactmap/), and on easy and hard targets from CASP12 and 13 [19, 20]. We chose some typical prediction methods implemented by DCA and machine learning for the comparison. State-of-the-art methods include CCMPred [2] (using DCA method), RaptorX-Contact [16] (based on double ResNet), and PconsC4 [21] (combination of ResNet and U-net). It should be noted that amino acid sequences in the test set have no similarity with the training set (at the 25% identity level) to prevent any overestimation of our predictor's performance, and that we used the same datasets for all four models.

In order to verify the effectiveness of the proposed neural network structure, we constructed three network structures by different combinations of ResNet and DenseNet, namely, 1D DenseNet + 2D DenseNet, 1D ResNet + 2D ResNet, and 1D DenseNet + 2D ResNet and compared them with our framework. The result is shown in the attached Table 1. We find the prediction accuracy by our network structure is higher than that by other three network structures.

To verify the validity of the proposed feature input, two other feature combinations (our feature combination without PSFM feature or PSSM feature) were designed for the experimental comparison. The result is shown in Table 2. We find the feature combination in the proposed method can obtain better accuracy than other two feature combinations.

**Figure 6:**
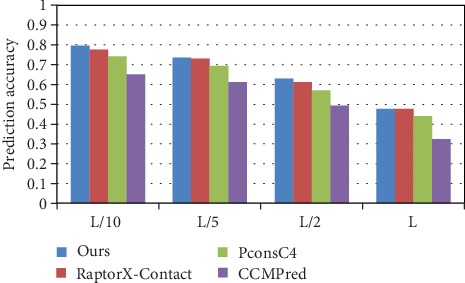
Comparison of method accuracy for long-range contact on PDB25.

The accuracy of the long-range contact predictions on the PDB25 dataset is illustrated in Figure 6, and the detailed prediction accuracies of the long-range contact in *L*/*k* (*k* = 10, 5, 2, and1) are shown in Table 3. Compared with RaptorX-Contact, our method has an increase of 1.9%, 0.4%, and 1.8% in *L*/*k* (*k* = 10, 5, and 2), with PconsC4 an increase of 5.5%, 4%, 5.9%, and 3.7% in *L*/*k* (*k* = 10, 5, 2, and 1), and with CCMPred an increase of 14.5%, 12.3%, 13.8%, and 15.4% in *L*/*k* (*k* = 10, 5, 2, and 1). We find that our prediction accuracy is better than that of PconsC4 and CCMPred in long-range contact and while our prediction accuracy in top *L*/*k* (*k* = 1) is like that of RaptorX-Contact, our prediction accuracy of top *L*/*k* (*k* = 10, 5, and 2) is higher. The prediction comparison of the medium and short-range contacts in *L*/*k* (*k* = 10, 5, 2, and 1) is also shown in Table 3.

**Figure 7:**
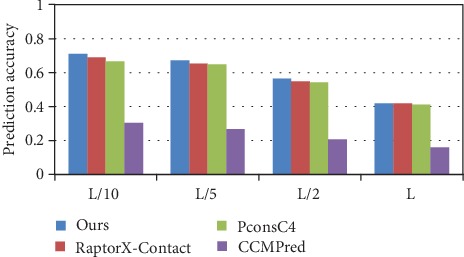
Comparison of method accuracy for long-range contact on 76 hard CAMEO.

The prediction accuracy of long-range contact on the 76 hard CAMEO test set is illustrated in Figure 7, and the detailed prediction results for different methods on the 76 hard CAMEO dataset are shown in Table 4. Compared with PconsC4, our method has an increase of 4.6%, 2.5%, 2%, and 0.9%, with RaptorX-Contact an increase of 2%, 2%, and 1.5% in *L*/*k* (*k* = 10, 5, and 2), and with CCMPred a significant increase for the accuracy of long-range contact in *L*/*k* (*k* = 10, 5, 2, and 1). The prediction comparison of the medium and short-range contact in *L*/*k* (*k* = 10, 5, 2, and 1) is also shown in Table 4.

**Figure 8:**
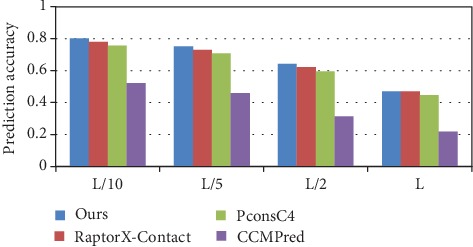
Comparison of method accuracy for long-range contact on Mems400.

For the Mems400 dataset, the long-range contact prediction accuracy is shown in Figure 8, and the detailed prediction results by different methods are shown in Table 5. Our model's prediction accuracy of the long-range contact in *L*/*k* (*k* = 10, 5, 2, 1) is 80.1%, 75.2%, 64.3%, and 47.1%, respectively. Compared with PconsC4, there is an increase of 4.5%, 4.4%, 4.7%, and 2.4%, with RaptorX-Contact an increase of 2.1%, 2.1%, 2%, and 0.1%, and with CCMPred there is also a significant increase for the accuracy of long-range contact in *L*/*k* (*k* = 10, 5, 2, and 1).

For the CASP12 dataset, we separate the long, medium and short contact results on the hard and easy CASP12 targets, which are shown in Table 6. We find the performance on the easy CASP12 targets is a little better than the hard CASP12 targets.

**Figure 9:**
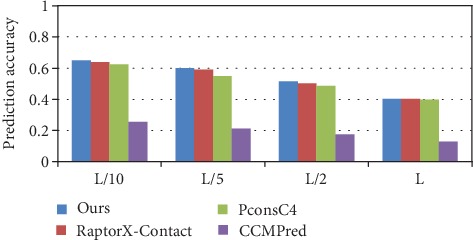
Comparison of method accuracy for long-range contact on the easy CASP12 targets.

The accuracy comparison of long-range contact prediction by different methods is illustrated in Figure 9, and Table 7 shows the detailed prediction results with long, medium and short contacts. For the long-range contact prediction, our accuracy in *L*/*k* (*k* = 10, 5, 2, and 1) is 64.9%, 60.1%, 51.4%, and 40.3%, respectively. Compared to PconsC4, there is an increase of 2.6%, 5.4%, 2.8%, and 0.6%, to RaptorX-Contact, the increase is about 1.0%, 1.2%, 1.2%, and 0.1% in *L*/*k* (*k* = 10, 5, 2, and 1) and with CCMPred, there is also a significant increase for the accuracy of long-range contact in *L*/*k* (*k* = 10, 5, 2, and 1). For medium contact and short contact, we find most of our accuracy results in *L*/*k* (*k* = 10, 5, 2, and 1) are better than PconsC4, RaptorX-Contact, and CCMPred.

For the CASP13 dataset, we divide the CASP13 targets into hard and easy targets which are shown in Tables 8 and 9. Besides, we separate the long, medium and short contact results on the hard and easy CASP13 targets which are shown in Table 10.

**Figure 10:**
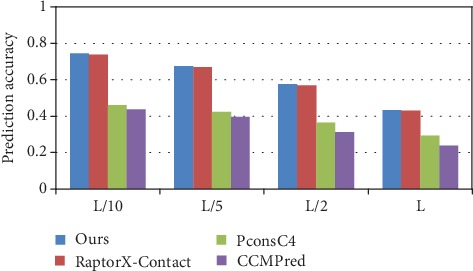
Comparison of method accuracy for long-range contact on the hard CASP13 targets.

The accuracy of the long-range contact predictions on the CASP13 dataset is illustrated in Figure 10, and the detailed prediction accuracies of the long-range contact in *L*/*k* (*k* = 10, 5, 2, and 1) are shown in Table 11. Compared with RaptorX-Contact, our method has an increase of 0.7%, 0.6%, 0.6%, and 0.1% in *L*/*k* (*k* = 10, 5, 2, and 1), with PconsC4 and CCMPred there is a significant increase for the accuracy of long-range contact in *L*/*k* (*k* = 10, 5, 2, and 1). The prediction comparison of the medium- and short-range contacts in *L*/*k* (*k* = 10, 5, 2, and 1) is also shown in Table 11. We find that our prediction accuracy is better than that of RaptorX-Contact, PconsC4, and CCMPred methods.

**Figure 11:**
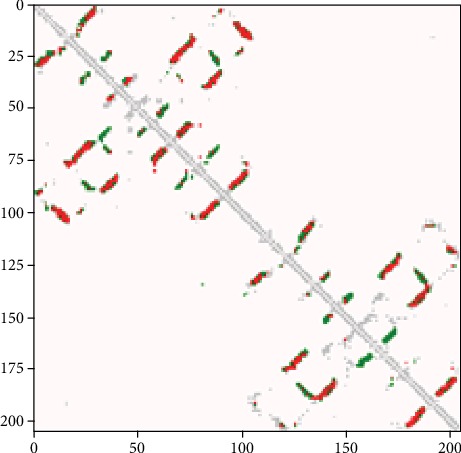
Predicted contacts of 5eo9B from the CAMEO dataset. Red (green) dots indicate correct (wrong) predictions, and silver dots indicate true contacts.

**Figure 12:**
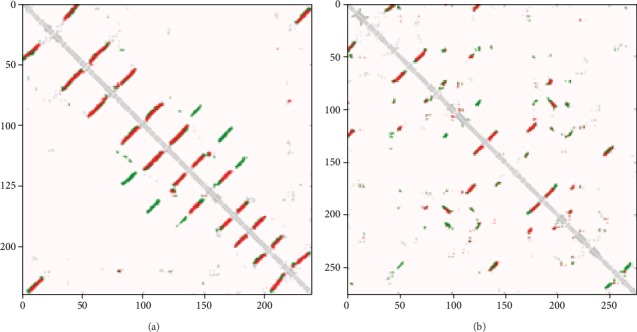
(a) Predicted contacts of 1qd6C from the Mems400 dataset. (b) Predicted contacts of T0944 from the CASP12 dataset. Red (green) dots indicate correct (wrong) predictions, and silver dots indicate true contacts.

**Figure 13:**
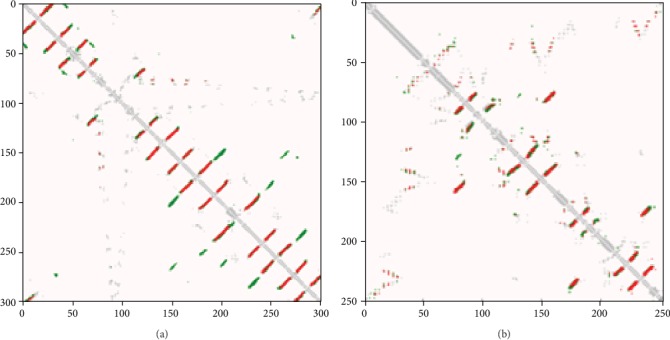
(a) Predicted contacts of 2porA from the Mems400 dataset. (b) Predicted contacts of 4xmqB from the CAMEO dataset. Red (green) dots indicate correct (wrong) predictions, and silver dots indicate true contact.

To further analyze the performance of our network framework, we made a comparison image of predicted contact and real contact for the protein sequences in the related test set. Figures 11–13 are the comparison chart between the prediction contact map and true contact map, where red (green) dots indicate correct (wrong) predictions and silver dots indicate true contacts. 5eo9B is a 206-residue long alpha-helix, beta-fold protein that binds to random curls released by the CAMEO dataset on 2016-01-06, and the correct (wrong) predicted contact and true contact of this protein is shown in Figure 11. From this figure, we can see that the overall majority of predicted contacts are correct.

We analyzed the contact prediction accuracy for other proteins in this way. 1qd6C is a 240-residue long protein with *β*-fold combining with random distortion released by the Mems400 dataset in 1999-10-25.  Figure 12(a) shows the correct (wrong) predicted and true protein contacts. T0944 is a 220-residue long alpha-helix, beta-folding protein that binds to random curling released by the CASP12 dataset.  Figure 12(b) shows the correct (wrong) predicted and true protein contacts. We also added the all-alpha and all-beta proteins to show the contact prediction accuracy by the proposed method. 2porA is a 301-residue long protein with all *β*-fold released by the Mems400 dataset. And 4xmqB is a 254-residue long protein with all *α*-helix released by the CAMEO dataset. Their correct (wrong) predicted and true protein contacts are shown in Figures 13(a) and 13(b). It can be seen that the proposed method is suitable for the contact map prediction of all-alpha or all-beta proteins. From these examples, we find that our method correctly predicted most contacts, and these improved contact map results are useful for the assisted structure prediction of proteins with various structures.

## 4. Conclusion and Future Work

In this paper, we have presented a prediction method for constructing protein contact maps using an integrated framework with ResNet and DenseNet. This method combines the advantages of ResNet's identity and residual mapping with DenseNet's dense connection and fully exploits them to help reduce the gradient disappearance problem and feature reusability, reduce the number of parameters, and capture the complex sequence-contact relationship and correlation between features. For the input feature, we have added a new position-specific frequency matrix feature (PSFM) besides the position-specific scoring matrix (PSSM), secondary structure (SS3), and 3-state solvent accessibility (ACC). These measures can effectively process sequential and pairwise features to predict the contact probability between residues and improve the prediction accuracy. The experimental results show that our proposed method is superior to other well-known methods. For easy implementation, all data used in this work and the source code for feature computing can be accessible at https://http://github.com/lnyile/Protein-Contact-Map-Rse_Dense.

While the accuracy of our model's top *L*/*k* (*k* = 10, 5, and 2) predictions was better than existing methods, the accuracy of top *L* prediction was not always significantly better. Combining more effective features as inputs and constructing a new deep learning neural network framework will further improve precision. Directions for future work include using the graphical representation and structural similarity of protein sequences to construct feature vectors as input for the deep learning framework to improve our model's predictions.

## Figures and Tables

**Table 1:** Comparison between our network structure and other ResNet and DenseNet combinations.

		Ours	1D ResNet + 2D ResNet	1D DenseNet + 2D DenseNet	1D DenseNet + 2D ResNet
Long	*L*/10	0.745	0.723	0.693	0.654
	*L*/5	0.675	0.654	0.627	0.597
	*L*/2	0.575	0.554	0.514	0.483
	*L*	0.432	0.421	0.415	0.386

Medium	*L*/10	0.742	0.721	0.701	0.675
	*L*/5	0.665	0.647	0.621	0.603
	*L*/2	0.568	0.541	0.529	0.487
	*L*	0.349	0.332	0.318	0.298

Short	*L*/10	0.722	0.703	0.672	0.634
	*L*/5	0.627	0.606	0.593	0.569
	*L*/2	0.464	0.438	0.417	0.396
	*L*	0.278	0.273	0.247	0.208

**Table 2:** Comparison between our feature input and other feature combinations.

		Ours	Ours without PSSM	Ours without PSFM
Long	*L*/10	0.745	0.718	0.698
	*L*/5	0.675	0.661	0.631
	*L*/2	0.575	0.557	0.512
	*L*	0.432	0.418	0.409

Medium	*L*/10	0.742	0.726	0.713
	*L*/5	0.665	0.641	0.627
	*L*/2	0.568	0.538	0.521
	*L*	0.349	0.337	0.315

Short	*L*/10	0.722	0.709	0.678
	*L*/5	0.627	0.611	0.585
	*L*/2	0.464	0.442	0.413
	*L*	0.278	0.271	0.259

**Table 3:** Long-, medium-, and short-range contact results by four different methods for PDB25.

		Ours	RaptorX-Contact	PconsC4	CCMPred
Long	*L*/10	0.796	0.777	0.741	0.651
	*L*/5	0.735	0.731	0.695	0.612
	*L*/2	0.631	0.613	0.572	0.493
	*L*	0.478	0.478	0.441	0.324

Medium	*L*/10	0.778	0.767	0.731	0.632
	*L*/5	0.674	0.667	0.634	0.593
	*L*/2	0.483	0.459	0.417	0.341
	*L*	0.295	0.296	0.259	0.193

Short	*L*/10	0.775	0.761	0.728	0.632
	*L*/5	0.646	0.635	0.617	0.514
	*L*/2	0.398	0.400	0.372	0.295
	*L*	0.238	0.240	0.229	0.192

**Table 4:** Long-, medium- and short-range contact results for 76 hard CAMEO.

		Ours	RaptorX-Contact	PconsC4	CCMPred
Long	*L*/10	0.711	0.691	0.665	0.304
	*L*/5	0.672	0.652	0.647	0.268
	*L*/2	0.563	0.548	0.543	0.207
	*L*	0.420	0.420	0.411	0.159

Medium	*L*/10	0.705	0.690	0.664	0.278
	*L*/5	0.632	0.614	0.598	0.227
	*L*/2	0.435	0.426	0.419	0.145
	*L*	0.278	0.278	0.271	0.103

Short	*L*/10	0.692	0.673	0.651	0.227
	*L*/5	0.592	0.578	0.543	0.163
	*L*/2	0.398	0.371	0.358	0.118
	*L*	0.239	0.235	0.231	0.097

**Table 5:** Long-, medium- and short-range contact results for Mems400.

		Ours	RaptorX-Contact	PconsC4	CCMPred
Long	*L*/10	0.801	0.780	0.756	0.523
	*L*/5	0.752	0.731	0.708	0.457
	*L*/2	0.643	0.623	0.596	0.313
	*L*	0.471	0.470	0.447	0.218

Medium	*L*/10	0.683	0.667	0.643	0.363
	*L*/5	0.555	0.539	0.502	0.268
	*L*/2	0.361	0.334	0.309	0.154
	*L*	0.220	0.220	0.215	0.109

Short	*L*/10	0.621	0.602	0.583	0.275
	*L*/5	0.483	0.468	0.435	0.193
	*L*/2	0.301	0.273	0.261	0.117
	*L*	0.160	0.159	0.154	0.089

**Table 6:** Long-, medium- and short-range contact results on the easy and hard CASP12 targets.

	Long				Medium				Short			
	*L*/10	*L*/5	*L*/2	*L*	*L*/10	*L*/5	*L*/2	*L*	*L*/10	*L*/5	*L*/2	*L*
Easy	0.649	0.601	0.514	0.403	0.675	0.577	0.403	0.255	0.662	0.551	0.321	0.273
Hard	0.642	0.596	0.503	0.394	0.583	0.564	0.395	0.249	0.653	0.543	0.315	0.272

**Table 7:** Long-, medium-, and short-range contact results on the easy CASP12 targets.

		Ours	RaptorX-Contact	PconsC4	CCMPred
Long	*L*/10	0.649	0.639	0.623	0.254
	*L*/5	0.601	0.589	0.547	0.211
	*L*/2	0.514	0.502	0.486	0.175
	*L*	0.403	0.402	0.397	0.129

Medium	*L*/10	0.675	0.665	0.659	0.253
	*L*/5	0.577	0.568	0.557	0.187
	*L*/2	0.403	0.395	0.381	0.139
	*L*	0.255	0.253	0.249	0.101

Short	*L*/10	0.662	0.651	0.643	0.252
	*L*/5	0.551	0.543	0.538	0.186
	*L*/2	0.321	0.314	0.301	0.127
	*L*	0.273	0.273	0.269	0.095

**Table 8:** PDB code and chain identification for CASP13 hard targets.

T0954-D1	T0957s1-D2	T0959-D1	T0960-D3	T0963-D3	T0964-D1	T0965-D1
T0966	T0979-D1	T0981-D1	T0981-D4	T0981-D5	T0982-D2	T0999-D2
T1011-D1	T1015s2-D1	T1021s1-D1	T1021s2-D1	T1022s1-D1	T1022s1-D2	T1022s2-D1

**Table 9:** PDB code and chain identification for CASP13 easy targets.

T0951	T0960-D5	T0961	T0962	T0963-D5	T0967	T0971
T0973	T0974s1	T0976	T0977	T0982-D1	T0983	T0984-D1
T0984-D2	T0993s1	T0993s2	T0995	T0999-D3	T0999-D4	T0999-D5
T1002	T1003	T1004-D1	T1004-D2	T1006	T1013	T1014
T1016	T1017s1-D1	T1018	T1019s2-D1			

**Table 10:** Long-, medium-, and short-range contact results on the hard and easy CASP13 targets.

	Long				Medium				Short			
	*L*/10	*L*/5	*L*/2	*L*	*L*/10	*L*/5	*L*/2	*L*	*L*/10	*L*/5	*L*/2	*L*
Hard	0.745	0.675	0.575	0.432	0.742	0.665	0.568	0.349	0.722	0.627	0.464	0.278
Easy	0.753	0.681	0.582	0.441	0.753	0.671	0.576	0.353	0.736	0.632	0.469	0.282

**Table 11:** Long-, medium-, and short-range contact results on the hard CASP13 targets.

		Ours	RaptorX-Contact	PconsC4	CCMPred
Long	*L*/10	0.745	0.738	0.460	0.437
	*L*/5	0.675	0.669	0.423	0.396
	*L*/2	0.575	0.569	0.363	0.313
	*L*	0.432	0.431	0.294	0.238

Medium	*L*/10	0.742	0.735	0.558	0.409
	*L*/5	0.665	0.662	0.524	0.327
	*L*/2	0.568	0.565	0.465	0.213
	*L*	0.349	0.348	0.377	0.145

Short	*L*/10	0.722	0.718	0.427	0.357
	*L*/5	0.627	0.621	0.373	0.289
	*L*/2	0.464	0.453	0.301	0.176
	*L*	0.278	0.278	0.216	0.124

## Data Availability

The data used to support the findings of this study are included within the article.
